# Gold Nanoparticle-Modified Molecularly Imprinted Polymer-Coated Pencil Graphite Electrodes for Electrochemical Detection of Bisphenol A

**DOI:** 10.1021/acsomega.4c07688

**Published:** 2024-12-25

**Authors:** Fatma Yılmaz, Nemah Abu Shama, Süleyman Aşır, Havva Çobanoğulları, Ercüment Yolaç, Aşkın Kiraz, Ilgım Göktürk, Adil Denizli, Deniz Türkmen

**Affiliations:** † Chemistry Technology Division, Vocational School of Gerede, Bolu Abant Izzet Baysal University, Bolu 14900, Turkey; ‡ Department of Medical Research, China Medical University Hospital, China Medical University, Taichung 404, Taiwan; § Research Center for Science, Technology and Engineering (BILTEM), Near East University, North Cyprus, Mersin 10, Nicosia 99138, Turkey; ∥ Department of Biomedical Engineering, Faculty of Engineering, Near East University, North Cyprus, Mersin 10, Nicosia 99138, Turkey; ⊥ Department of Biological Sciences, Faculty of Arts&Sciences, Eastern Mediterranean University, North Cyprus, Mersin 10, Famagusta 99628, Turkey; # Ataturk Faculty of Education Nicosia, Near East University, North Cyprus, Mersin 10, Nicosia 99138, Turkey; ∇ Department of Chemistry, Faculty of Science, Hacettepe University, Beytepe, Ankara 06800, Turkey

## Abstract

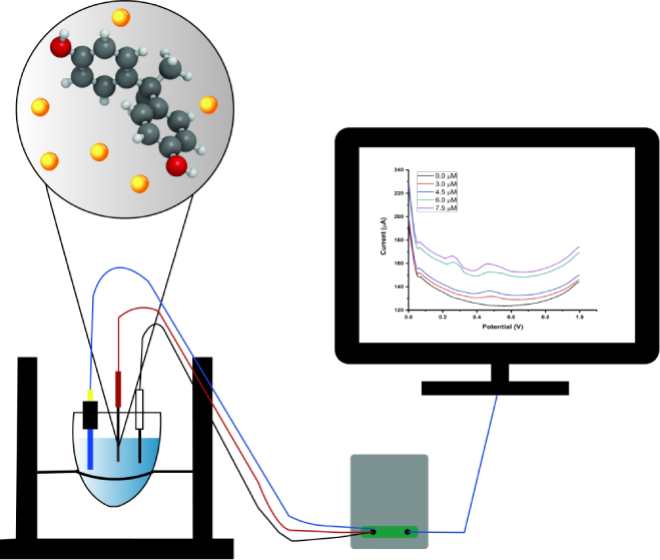

The sensitive Bisphenol A (BPA) detection by an electrochemical sensor based on gold nanoparticle-doped molecularly imprinted polymer was successfully improved. This study describes the development of a method for BPA detection in both aqueous solution and real water samples using N-methacroyl-(L)-cysteine methyl ester and N-methacryloyl-(L)-phenylalanine methyl ester coated pencil graphite electrodes modified with AuNPs by differential pulse voltammetry (DPV). Importantly, AuNPs, which increase the electroactivity, were used to increase the surface area of a BPA-imprinted pencil graphite electrode (MIP PGE) sensor. Scanning electron microscopy and spectrophotometry analysis were used for the characterization. The DPV response of the synthesized electrode showed distinguished electrical conductivity. The MIP PGE and nonimprinted pencil graphite electrode (NIP PGE) sensor were evaluated for selective and sensitive detection of BPA in aqueous solutions. Five different BPA concentrations (1.5, 3.0, 4.5, 6.0, and 7.5 μM) were applied to the MIP PGE, and the DPV recognized signal responses with a correlation coefficient value of 0.9965. The modified electrode demonstrated good electrocatalytic activity toward BPA for the linear concentration range of 1.5–7.5 μM, and a low limit of detection was found as 0.1610 μM. The results show that the MIP PGE sensor has excellent potential for selective and sensitive detection of BPA in real water samples.

## 1 Introduction

Bisphenol A (4,4′-(propane-2,2-diyl)diphenol, BPA) is an organic and synthetic material commonly used as a monomer to obtain polymeric substances such as epoxy, polyester, polysulfone resins and polycarbonate.^1,2^ For example, polycarbonate, which is among these polymers, is mainly used in industry to produce plastic bottles, food containers, packaging materials, etc.^3,4^ Additionally, BPA can be found in commonly used items such as medicals, sunglasses, adhesives and powder coatings.^5,6^ Estimates suggest that over 100 tons of BPA are released into the environment annually.^7^ Moreover, due to its structural resemblance to vital hormones like diethylstilbestrol and estradiol, BPA is classified as an endocrine disruptor. Because of its structural similarity to estrogen hormone, it can exert its effects by binding to estrogen receptors and disrupting the normal binding mechanism of estrogen hormone.^8^ Studies have demonstrated that even very low doses of BPA can affect the function of various organs in the human body. These organs include the brain, ovaries, thyroid, reproductive organs and immune system.^9^ In recent studies, BPA has been shown to be involved in the pathogenesis of different diseases, such as behavioral disorders, cardiovascular diseases, obesity, diabetes, neurotoxicity and hepatotoxicity.^10,11^ According to the scientific evidence evaluated by the European Food Safety Authority’s experts on the safety of BPA, the tolerable daily intake of BPA was set at 4 μg kg^–1^ per body weight/day.^12^ The permissible migration levels of BPA do not exceed 0.6 mg/kg in China and 0.5 mg/kg in Europe.^13,14^

As the negative effects of BPA exposure have been described in many studies, determining the concentration of BPA that can contaminate water, beverages and food has become very important. Various methods such as fluorimetry,^15^ molecular suppression,^16^ enzyme-linked immunosorbent assay,^17^ gas chromatography,^18^ high performance liquid chromatography^19^ and electrochemical methods^17,20^ have been used to determine BPA concentration. Importantly, the electropolymerization method has gained importance in the development of electrochemical sensors and biosensors due to its advantages such as good repeatability, selectivity, modification of the electrode, low cost, stability and electrochemical recycling.^21,22^ Numerous studies have been conducted in the literature on the use of modified electrodes for the quantitative and qualitative determination of BPA.^17^ Using a sensitizer, a highly sensitive photoelectrochemical aptasensor was developed to detect BPA by enhancing the photoactivity of CdS/Ni-MOF. While the sensor shows excellent performance, selectivity, and versatility, it has some drawbacks, including complex preparation and a relatively lengthy detection process.^23^ A graphitic carbon-coated PtCoNi alloy was prepared through high-temperature pyrolysis. The carbon coating improved the catalytic stability. The sensor showed excellent catalytic activity, with a wide detection range of 2.0–140.0 μM and a low detection limit of 0.19 μM for BPA.^24^ Besides, a sensitive sensor for BPA detection was developed using 4-MBA/gold nanodendrites/GCE, offering a wide linear range from 0.05 to 55 μmol L^–1^ (R^2^ = 0.995), a low detection limit of 1.2 nmol L^–1^, and strong anti-interference capability.^25^

Molecularly imprinted polymers (MIPs) are polymeric matrices produced by imprinting technology. They can mimic natural recognition systems such as enzymes, substrates, antibodies, and antigens. MIPs are primarily utilized for separating and analyzing environmental samples and biological macromolecules due to their high specificity and selectivity.^26−28^ In particular, the MIPs are enriched with unique functional chemical groups and imprinted sites that are highly specific and selectively recognize template molecules. The synthesis of MIPs relies on the template-activated formation of complementary detection cavities. This process gives MIPs a specific size and shape, making them functional for a biological or chemical target molecule (the template molecule).^27,29^ These enhancements can be beneficial in the selective adsorption or separation of desired molecules.^30,31^ Moreover, it has been shown that MIPs can be used as specifically constructed sorbents that exhibit high selectivity toward a particular structure. One study emphasized the importance of achieving a low limit of detection (LOD), especially when analyzing highly complex matrices. The same study also suggests a selective extraction method for the compound under investigation could overcome this challenge.^32^ Several methods have been used efficiently so far to develop highly selective sorbents using MIPs.^33−35^ The intrinsic properties of MIPs, such as their uniquely designed binding sites, simplicity, affordability and stability, have led to their widespread use across many fields, such as drug delivery and catalysis, biosensors and sensors, separation processes, and purification.^28^ Based on the findings from different studies, sensor methods have proven to be the most suitable and critical approach for BPA detection compared to other methods. In particular, MIP-based and aptamer-based sensors have been shown to be very effective in detecting BPA.^7^ MIPs are examples of sensor recognition materials that can adequately reduce interference from other similar analytes in terms of functional groups and structures.^2^ In one study, an MIP electrode, more specifically, electrochemically reduced graphene oxide coated with a glassy carbon electrode, was used to determine BPA in real and standard samples by differential pulse voltammetry (DPV). The correlation coefficient was determined to be 0.99, and the detection limit was determined to be 0.2 nmolL^–1^ (S/N = 3).^36^ In another study, CV and DPV approaches were used to investigate the electrochemical MIP sensors’ electrochemical behavior.^26,37^ The detection limit of the imprinted electrochemical sensor toward BPA was determined to be 3.2 × 10^–12^ mol L^–1^ (S/N = 3). In addition, BPA was detected by electrochemical MIP sensors in actual plastic samples with good recoveries differing from 93.3–103.0%.^10^

Recently, nanotechnology, which uses various nanomaterials to develop specific sensors, has aimed to improve the sensitivity of current tests. Although various studies are still ongoing, nanotechnology has shown promising results in the analysis of BPA. When MIPs are used together with nanomaterials, it has been shown that the sensitivity and specificity increase significantly because they have a large surface area.^7^ In addition, bulky polymeric microparticles have often been observed when using conventional methods to develop MIPs, which is a major problem when integrating them into a sensor or other applications such as drug delivery and medical imaging. It is well established that nanoparticles (NPs) have some properties that make them advantageous compared to bulk matrices. For example, they are easier to immobilize, have higher surface area to volume ratio, they are soluble and more diffusible, and have a higher adsorption efficiency. Therefore, their potential use can be increased if NPs are combined with molecular imprinting.^29,38^ In recent years, the use of MIP-NPs has been widely observed in various fields such as liquid chromatography,^39^ capillary electrochromatography,^40^ drug delivery^29^ and sensors.^29,41^

The electrochemical properties of BPA have been investigated using different voltammetric electrodes, including MIPs, semipermeable-based membranes, and conductive membranes that have polymeric properties. In the literature, in many studies, MIPs and nanomaterials have been shown to recognize and bind to BPA selectively and sensitively, which makes them highly attractive tools in monitoring the analytical and environmental samples. Nevertheless, NPs are costly, and the preparation of electrodes consists of several steps.^4,42^ Although some satisfactory findings have been obtained, more sensitive, less complex and rapid electrochemical approaches should be investigated and developed.

This study aims to sensitively and selectively determine BPA using the MIPs in electrode cell systems. Although several studies have already been performed on modified MIP sensors, in this study, N-methacroyl-(L)-cysteine methyl ester and N-methacryloyl-l-phenylalanine methyl ester coated pencil graphite electrodes modified with AuNPs were used to increase the electron transfer mechanism and achieve higher sensitivity to BPA molecules. The parameters that affect the electrochemical response, such as pH and competitors were examined by DPV.

## 2 Experimental Studies

### 2.1 Materials and Methods

Gold nanoparticles (40 nm in diameter) were purchased from SPI Supplies Inc. (West Chester, PA, USA). 2-Hydroxyethyl methacrylate (HEMA), ammonium persulfate (APS), ethylene glycol dimethacrylate (EDMA), bisphenol A (BPA), (±)-phenol, 1-naphthol, poly(vinyl alcohol) (PVA), sodium dodecyl sulfate (SDS), sodium bicarbonate (NaHCO_3_), sodium bisulfite (NaHSO_3_), and sodium hydroxide (NaOH) were obtained from Sigma-Aldrich Chemical Co. (St. Louis, Missouri, USA). Barnstead’s ROpure LP reverse osmosis unit (Dubuque, Iowa, USA) was used to purify and use the water in the experiments.

### 2.2 Synthesis of MIP and NIP Nanoparticles

First, N-Methacryloyl-(L)-cysteine methyl ester (MAC) and N-methacryloyl-(L)-phenylalanine methyl ester (MAPA) functional monomers were synthesized according to our previous study.^35^ Then, the complexation of MAC with (0.1 mmol: 0.01 nmol) was mixed for 1 h to determine the complex formation between MAC monomer and AuNPs, according to our previous study.^43^ Also, the MAPA-BPA precomplex was prepared by mixing the functional MAPA monomer and BPA (0.1 mmol: 0.01 mmol). BPA-imprinted NPs were synthesized according to the procedure as follows. A two-phase mini-emulsion process prepared MIP NPs. The monomer phase, consisting of HEMA (0.10 mmol) and EDMA (0.10 mmol), was added to phase II, which contained PVA (0.05 g) and SDS (0.05 g). The miniemulsion was obtained by homogenizing (T10, Ika Labortechnik, Germany) at 25000 rpm for 30 min. Then, the miniemulsion solution prepared previously was poured into phase I, which contains PVA (0.1 g), SDS (15 mg), and NaHCO_3_ (12 mg). The final mixture was placed in a polymer reactor (Radleys Carousel 6, Essex, UK), and after the mixture reached a temperature of 40 °C, the initiator pair of NaHSO_3_ and APS (1 mmol: mmol) was added. Polymerization was allowed to occur at 600 rpm for 24 h. The same procedure was applied to synthesize nonimprinted NIP NPs without adding BPA molecules. The NPs were washed several times with ethanol and deionized water to remove unreacted chemicals after polymerization by an ultracentrifuge (Allegra-64R Beckman Coulter, USA).

### 2.3 Preparation of PGE Sensors

The surface of the PGE was cleaned with 10 mL of pure ethyl alcohol and deionized water and then dried at room temperature before the coating of NPs. The NPs were drop-cast on the surface of PGE and fixed by UV radiation for 30 min. Methanol: acetic acid mixture (9:1 v/v) was used as a desorption agent to remove the template BPA molecule. The same method was used to create the nonimprinted NIP PGE sensor, but BPA was not added as the target molecule. BPA-imprinted MIP PGE and nonimprinted NIP PGE sensors were compared to evaluate the imprinting efficiency.

### 2.4 Characterization Studies

Dynamic light scattering (DLS, NanoS, Malvern Instruments) (*n* = 3) was used to determine the size distribution of the polymeric NPs. The surface morphology of NPs was also characterized by scanning electron microscopy (SEM, JSM-6400, JEOL, Akishima, Tokyo, Japan) to evaluate surface morphology after the samples were coated with a thin gold–palladium alloy coating. A (FTIR-ATR) spectrophotometer (Thermo Fisher Scientific, Nicolet iS10, Waltham, MA, USA) was used to characterize the MIP and NIP PGE sensors. The total reflectance was determined for 400 and 4000 cm^–1^. In addition, the surface characterization of MIP PGE and NIP PGE sensors was carried out by taking ten measurements from different surface parts using the sessile drop method with the Kruss DSA100 device (Hamburg, Germany). Energy Dispersive X-ray Analysis (also referred to as EDX) combined to Scanning Electron Microscopy (SEM) instrument (SEM-EDS; Tescan Model: GAIA3 + Oxford XMax 150 EDS), where the imaging capability of the microscope identifies the specimen of interest was used to obtain detailed high resolution images and elemental identification of the MIP PGE and nonimprinted NIP PGE sensors. Elemental identification and quantitative compositional information on MIP PGE and NIP PGE sensors were also evaluated by SEM-EDS. MIP PGE, and NIP PGE sensors/polymers sputtered onto the SEM stubs were evaluated for scanning process.

## 3 Results and Discussion

### 3.1 Characterization Analyses

**Figure 1:**
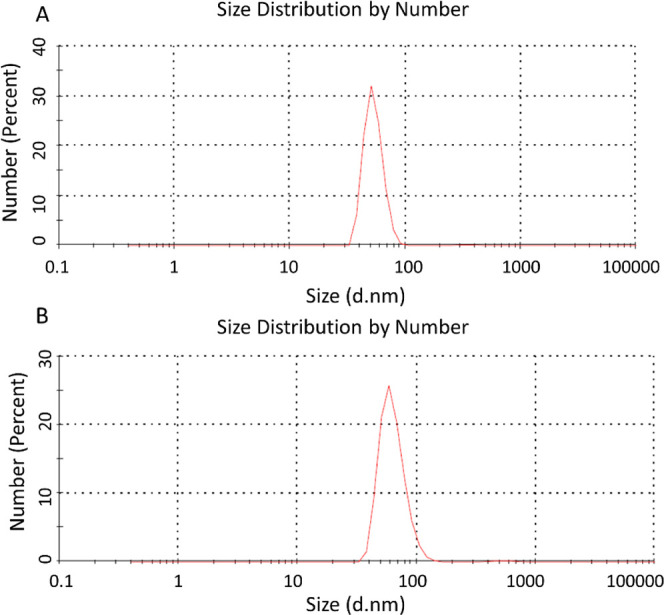
DLS analyses of NPs: A) NIP NPs B) MIP NPs.

**Figure 2:**
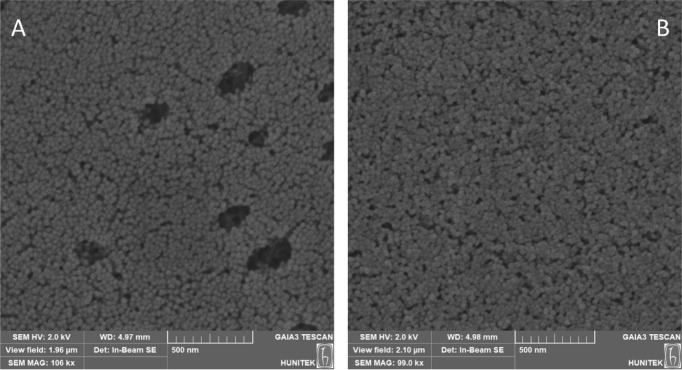
SEM images of NPs: A) NIP NPs B) MIP NPs.

MIP and NIP NPs synthesized by miniemulsion polymerization were characterized using a ZetaSizer analyzer and SEM. According to ZetaSizer results, MIP NPs have an average diameter of 63.43 nm (PDI: 0.076), while NIP NPs have an average diameter of 53.42 nm (PDI: 0.070). The results showed that molecular imprinting increased the hydrodynamic size of NPs (Figure 1). Molecular imprinting is a process that typically creates a polymer layer around a template molecule. This polymer coating increases the overall diameter of the nanoparticle, as the imprinting layer adds to the core size of the nanoparticle. Nanoparticles that are imprinted with molecular cavities may have a rougher surface, thus resulting in a larger apparent size measured in a solution. According to SEM images, MIP NPs and NIP NPs have uniform sizes and are spherical in the range of 50–60 nm (Figure 2).

**Figure 3:**
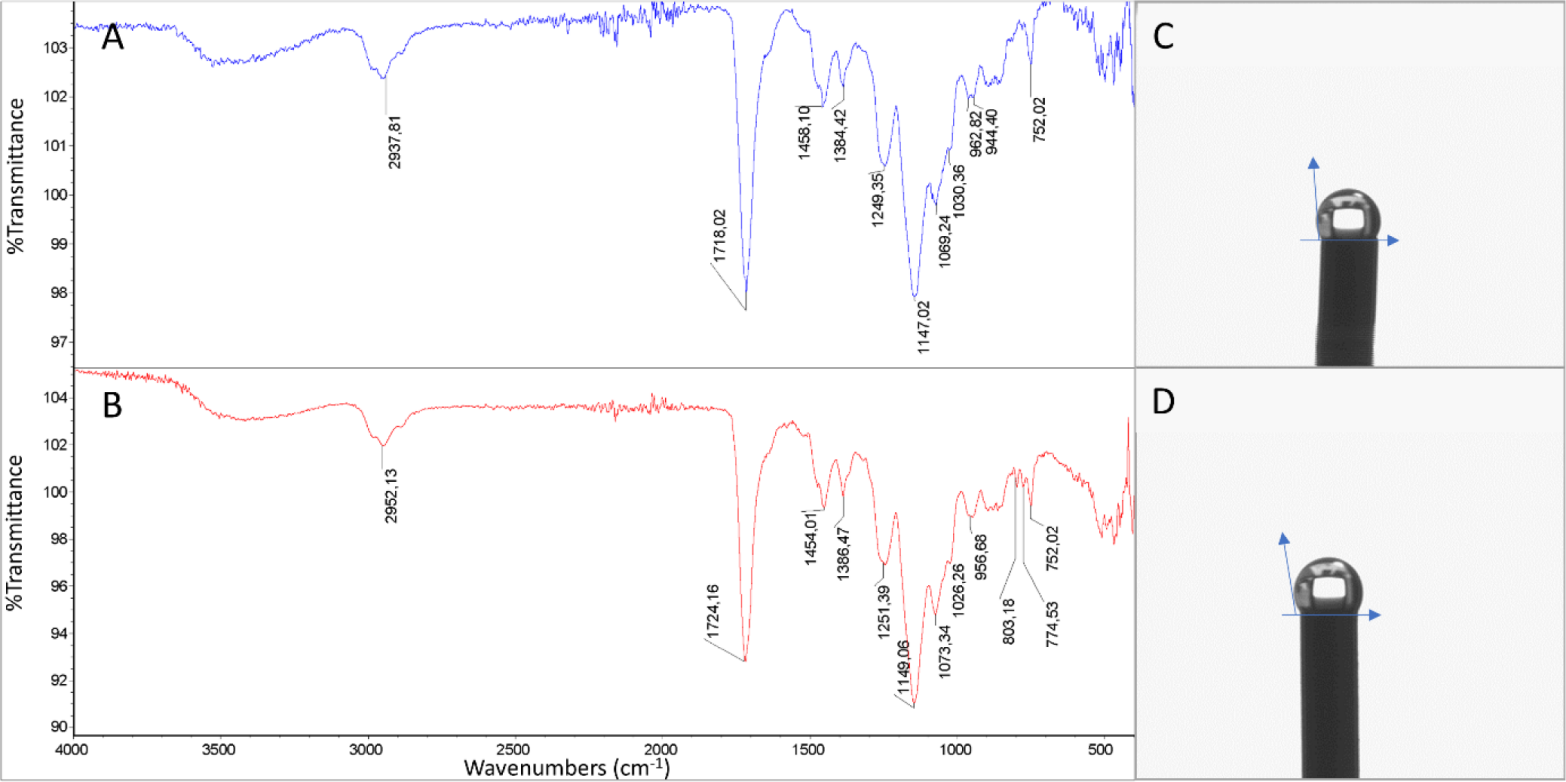
FTIR-ATR spectra of sensors. A) NIP PGE sensor, B) MIP PGE sensor; contact angle images of sensors C) NIP PGE sensor, D) MIP PGE sensor.

In the FTIR-ATR spectrum, the peaks around 2950 cm^–1^ suggest N–H stretching vibrations, which can be attributed to the amide group in the MAPA monomer. Other notable peaks are around 1720 cm^–1^, which corresponds to the stretching vibration of the C=O bond in the amide group of the MAPA monomer. Besides, the peaks around 1250 cm^–1^ suggest the stretching vibration of the C–O bond in the ester group of the MAPA monomer (Figure 3A). Furthermore, the peak observed around 774 cm^–1^ in the MIP PGE can be assigned to the stretching vibration of the C–OH bond of the BPA template molecule, which indicates the molecular imprinting was achieved successfully (Figure 3B). Besides, the contact angle measurement (Kruss DSA100) was used to investigate the surface-wetting properties of the PGE sensors. Figure 3C and D represent the CA images of the NIP PGE and MIP PGE sensors. The estimated CA values were calculated at 123.8° ± 1.2 for the NIP PGE (Figure 3C) and 129.5° ± 0.4 for the MIP PGE (Figure 3D) sensor. The contact angle values calculated by the DSA2 software revealed that the BPA coordination with the MAPA monomer enhanced the hydrophobicity of the MIP PGE sensor surface.

**Figure 4:**
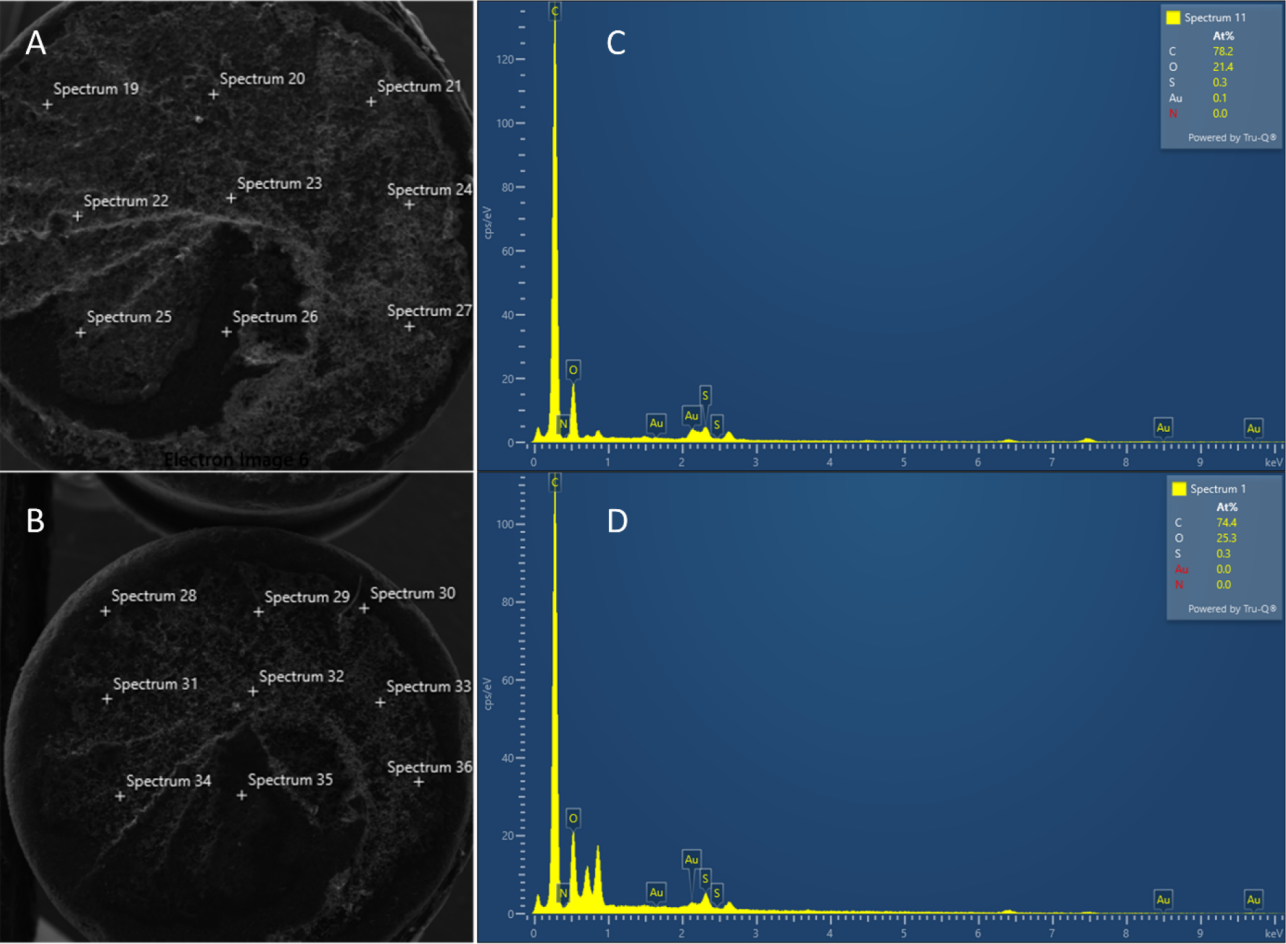
SEM and EDS images of (A, C) MIP-PGE, (B, D) NIP-PGE sensors, respectively.

Scanning Electron Microscopy (SEM) with Energy Dispersive X-ray Analysis (EDX) testing technique (SEM-EDX) provided detailed high resolution images of the MIP-PGE and NIP-PGE SPR sensors by rastering a focused electron beam across the surface and detecting secondary or backscattered electron signal. An Energy Dispersive X-ray Analyzer (EDX or EDS) was also used to provide elemental identification and quantitative compositional information. Figure 4. shows both SEM and EDS images for (A, C) MIP-PGE and (B, D) NIP-PGE, sensors, respectively. As reported from the figure, there are bands belonging to AuNPs and MAC monomers included in both MIP-PGE and NIP-PGE sensors. And the amount of S, Au elements due to the MAC monomer and particles present in the MIP-PGE and NIP-PGE sensors is clearly shown in the SEM-EDX images. Other values are not proportional to the amount of elements included in the data that can not be obtained from a depth greater than 100 nm below the surface.

### 3.2 Optimization and Kinetic Analysis by MIP PGE Sensor

**Figure 5:**
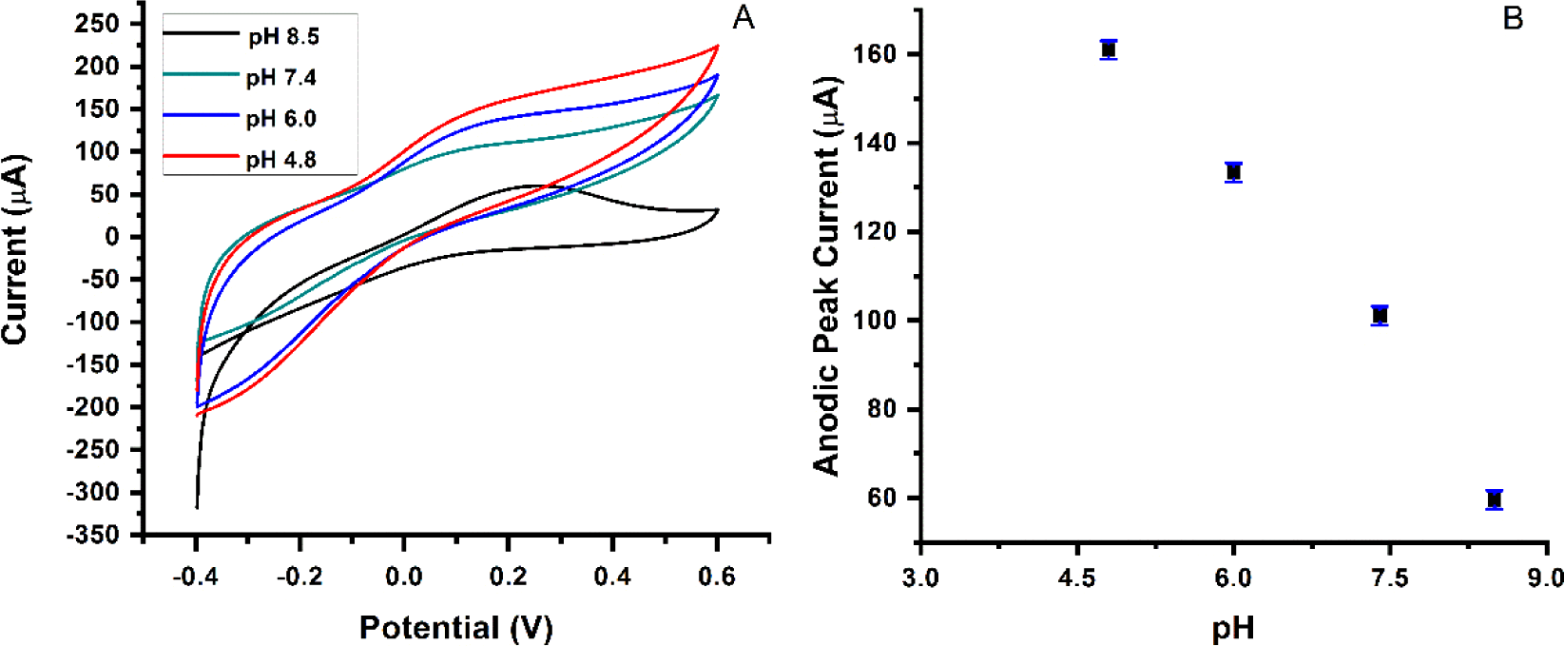
Optimization of the working pH for BPA by CV method in different buffers at room temperature. (A) The detection of BPA (8.323 μM) in different pH buffer solutions; (B) pH values vs the anodic peak current obtained.

The effect of pH on electrochemical activity has been demonstrated.^44^ Bisphenol A has a p*K*_a_ = 10.6, in aqueous solutions, the pH value is <10.6, while in molecular forms, the pH value is >10.6, and it is presented as HBPA^–^ or as BPA^2–^.^45^ Here, the effect of pH was optimized to enhance the electrochemical response of the modified electrodes for BPA, therefore, different buffers with various pH values (pH = 8.5 CHES; pH = 7.4 Tris; pH = 6.0 MES; and pH = 4.8 acetate buffer) were prepared. Figure 5 shows the detection of 8.323 μM BPA at different pH values, followed by the response of the voltammetric technique used with PGE 0.5/HB as WE in the electrochemical cell.

**Figure 6:**
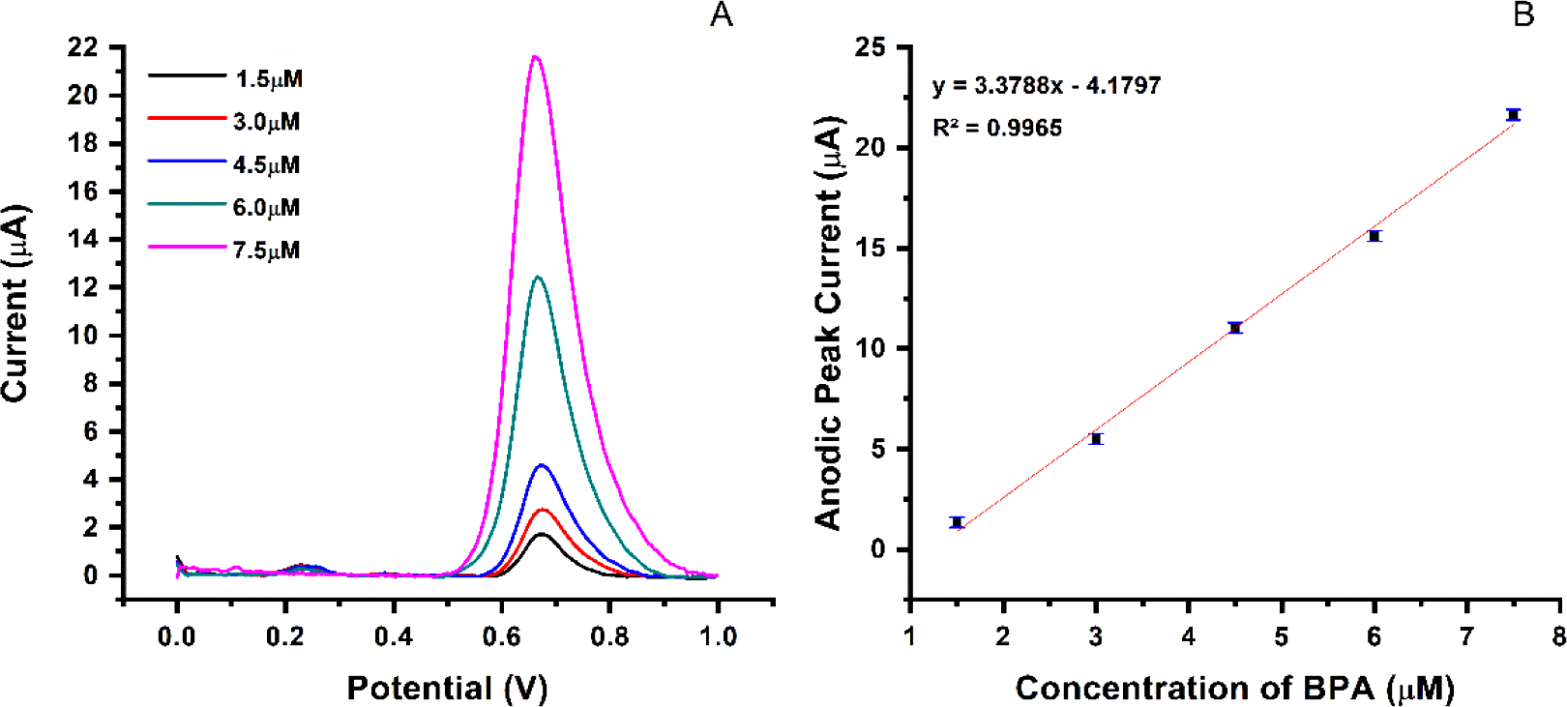
Optimization of BPA and kinetic parameters. (A) Obtained of BPA by DPV by MIP-PGE sensor; (B) calibration curve of BPA in concentrations ranges from 1.5 μM to 7.5 μM.

In CV, the highest voltammogram peak was detected at pH 4.8 compared to other pH values. This indicates that the amount of hydronium ions is exceptionally critical for the oxidation of BPA. Furthermore, in Figure 6, different concentrations of BPA were prepared from the stock solution for the calibration curve, ranging from (1.5 μM-7.5 μM) by dissolving a proper amount of BPA crystals in 1 mL of methanol as a solvent and continuing to the mark by adding acetate buffer solution (pH= 4.8), which was stored in the refrigerator at 4 °C after use. Kinetic analysis was performed using the MIP PGE sensor with five different concentrations of BPA (1.5, 3.0, 4.5, 6.0, 7.5 μM) in 0.1 M acetate buffer solution at 4.8 pH. The concentrations were detected by DPV technique with start and stop potentials of 0 and 1.0, respectively, in a modulation time of 0.05 s. In addition, the desorption solution (methanol: acetic acid (9:1 v/v)) was used between each measurement to desorb the BPA molecule from the MIP PGE sensor cavities to increase the electrode affinity. The measurements were repeated three times (*n* = 3) to calculate RSD% (the relative standard deviation) and repeatability accuracy.

### 3.3 DPV Response of the MIP PGE Sensor for Different BPA Concentrations

The modified electrode surface of the polymer, which has a negative charge, plays a crucial role in adsorbing BPA molecules, which typically have a positive charge. This interaction enhances the electrode’s sensitivity to the desired chemical species. The presence of amino acid phenylalanine in the MAPA monomer is significant in imparting this negative charge to the modified polymer electrode surface. Phenylalanine contains an acidic carboxyl group (−COOH) and a basic amino group (−NH_2_), resulting in its ability to act as a charge carrier. Phenylalanine has a pI value of 5.48 (p*K*_a1_: 2.58 and p*K*_a2_: 9.24). An increase in the pH value in the mobile phase leads to an increase in the deprotonation of the acid functional groups. Figure 6 shows the voltammogram results of four different BPA concentrations (1.5 μM-7.5 μM) measured by DPV, a more sensitive technique compared to other analytical techniques. In last five decades, electroanalytical analyses have been performed by different voltammetric techniques including differential pulse voltammetry (DPV), cyclic voltammetry (CV), linear sweep voltammetry (LSV) and square wave voltammetry (SWV).^46^ These voltammetric techniques are used in many areas such as environmental monitoring (analysis of pollutants or heavy metals),^47^ clinical analysis^46^ and in chemical/biosensors.^48^ It is highly critical to select the appropriate technique to detect the particular analyte with lowest limit of detection (LODs) and optimum sensitivity. Although CV has been used to determine formal potential, electron transfer kinetics and redox mechanisms, in recent years pulse techniques such as DPV are often used in the development of biosensors and electrochemical sensors due to their higher sensitivity.^46^ The low capacitive current observed with DPV makes this method more advantageous as it produces a high sensitivity. In addition, since the small step sizes in DPV result in narrower voltammetric peaks, it is possible to distinguish analytes with similar oxidation potentials.^46,49^ The potential peak was observed approximately at ∼0.7 V, where an increase in BPA concentration enhanced the oxidation of BPA. The relationship between the oxidation signal and the BPA concentration makes it possible to measure the BPA content quantitatively. Critically, the coefficient, which was determined to be 0.9965, as illustrated in Table 1, indicates a strong linear relationship between the response signal and the BPA concentration. Furthermore, the calibration curve with a high resolution for the voltammetric detection of BPA was shown in Figure 6B. The slope of the curve (2.981 × 10^–6^) is in the acceptable range for an electrochemical sensor, which underlines the sensitivity of the detection of BPA at low concentrations, which is exceptional compared to other studies,^50^ as shown by the determined LOD and LOQ values of 0.1610 and 0.4834 μM, respectively. In addition, a standard deviation (SD) of 1.441 × 10^–7^ was determined based on the calculations, showing acceptable reproducibility and accuracy. These results show that the MAPA monomer can increase the selectivity of the electrode for BPA molecules. These results indicate that the electrochemical detection method synthesized in this study is specific to BPA and could be used to measure BPA concentrations with increased sensitivity.

**Table 1:** Figures of Merit of BPA Detection by DPV

Method	aR^2^	bm (μA/μM)	cSD (μA)	dLR (μM)	eLOD (μM)	fLOQ (μM)
DPV	0.9965	2.981 × 10^–6^	1.441 × 10^–7^	1.5–7.5	0.1610	0.4834

a Correlation coefficient.

b Slope.

c Standard deviation.

d Linear range.

e Limit of detection.

f Limit of quantification.

### 3.4 Estimation of Selectivity and Imprinting Efficiency

Recently, integrating MIPs into sensors that act as biological recognition elements has attracted a lot of attention. Importantly, MIPs fabricated using imprinting technology act as a recognition element that selectively recognizes molecules by a template molecule in a complex matrix.^26,51^ An electrode coated with MIP plays a role in differentiation, creates unique cavities, and has an important interaction with the template molecule in terms of size and shape. These cavities are crucial for selectivity and increasing the efficiency of analyte binding. Furthermore, it has been shown that MIPs are physically and chemically stable structures. In addition, they have several properties that make them remarkable, e.g., they are very resistant to alkali solutions and acids, they can be easily synthesized, they are resistant to high pressure and high temperatures, they have a long lifetime and can be recycled and reused. In short, polymerization occurs around precomplex structures containing a template molecule by adding cross-linkers and initiators. Specific three-dimensional shapes are then created by removing the template. The interaction between imprinted sites and the template can be observed several times without affecting the performance of the imprinted sites. Particularly, the imprinting sites of the MIPs is highly selective and capable of increasing efficiency in rebinding targeted molecules.^52^ Due to these diverse properties, MIPs can be used to develop promising, durable and effective electrochemical sensors.

**Figure 7:**
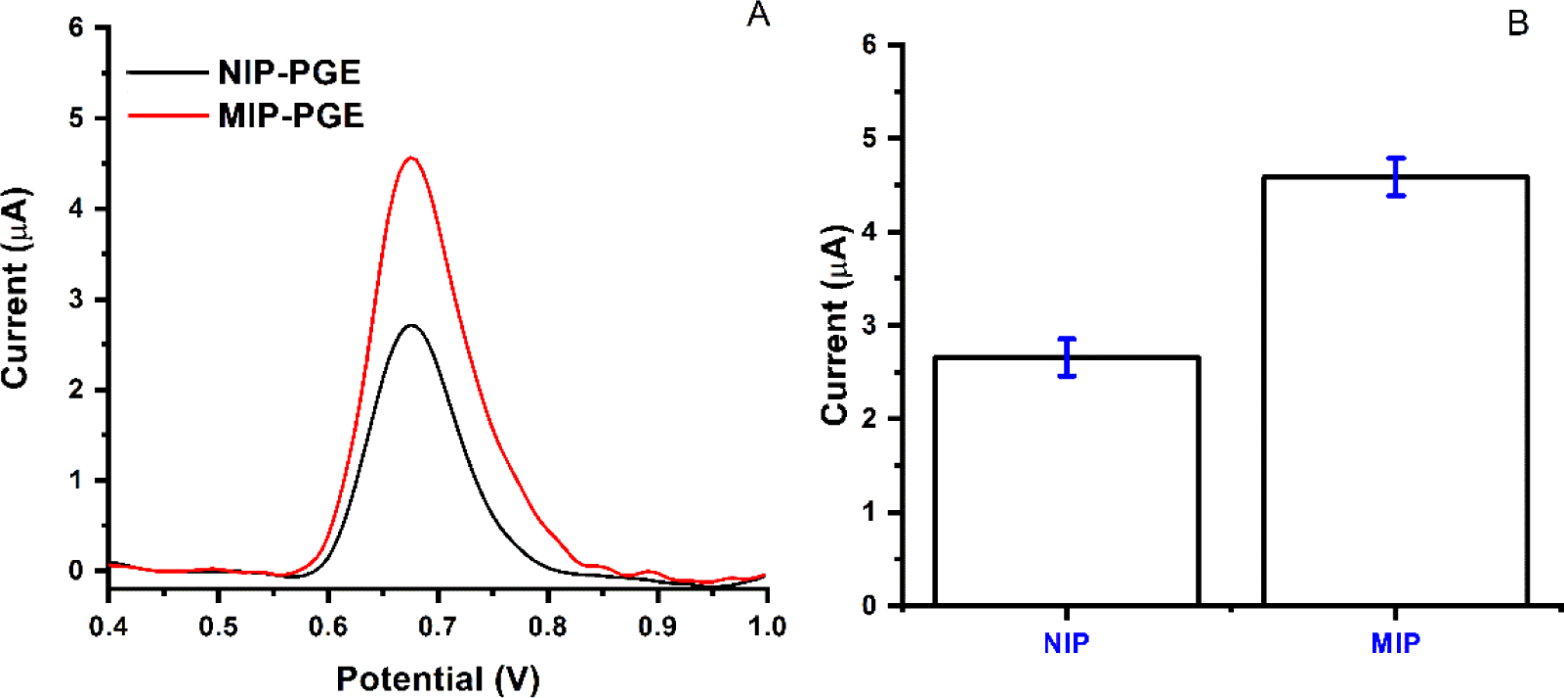
Response of PGE sensors. (A) MIP-PGE and NIP-PGE sensor responses for the BPA analyte; (B) bars representing the responses of MIP-PGE and NIP-PGE sensor (*n* = 3).

The production and recycling of BPA often result in environmental contamination with BPA, which seriously affects the ecosystem.^53^ There has been a growing interest in the selective and sensitive detection of BPA and monitoring it in food, biological and environmental samples due to its adverse effects on human health and ecology.^44,53^ Although several methods have been developed to detect BPA, a technique with several advantages, such as high selectivity, high sensitivity, low cost and simplicity is required to detect this chemical in complex matrices.^44^Figure 7 illustrates the evaluation of BPA detection using the MIP PGE and NIP PGE. The response of the electrodes for BPA indicates that the sensitivity of the electrodes is high. The response of MIP PGE and NIP PGE for BPA detection (3.0 μM) was compared, and the imprinting efficiency demonstrated that the developed sensor indicates remarkable selectivity toward BPA than competitor molecules. The obtained signals from two electrode sensors for BPA (3.0 μM) were illustrated in Figure 7A. Furthermore, the efficiency of the MIP PGE sensor in detecting BPA molecule (3.0 μM) was compared to that of the NIP PGE sensor, as shown in Figure 7B.

Moreover, several studies have been conducted in the literature to determine the concentration of BPA in water samples.^54−56^ Different studies have obtained different results regarding the BPA concentration in water samples. However, it was established that this compound is toxic to humans, even in very small quantities. Besides, since various interfering compounds may be present in the matrix, developing a technique to detect the desired chemical selectively is necessary. Therefore, a simple, effective and profitable method for determining BPA is essential. To test the selectivity of the MIP PGE sensor, other analogous structures, such as phenol and naphthol, which are structurally similar at the molecular level, were chosen as competitors. In detail, the hydroxyl group (−OH) is found to be a functional group in these three phenolic structures, which makes them structurally similar at the molecular level.

**Figure 8:**
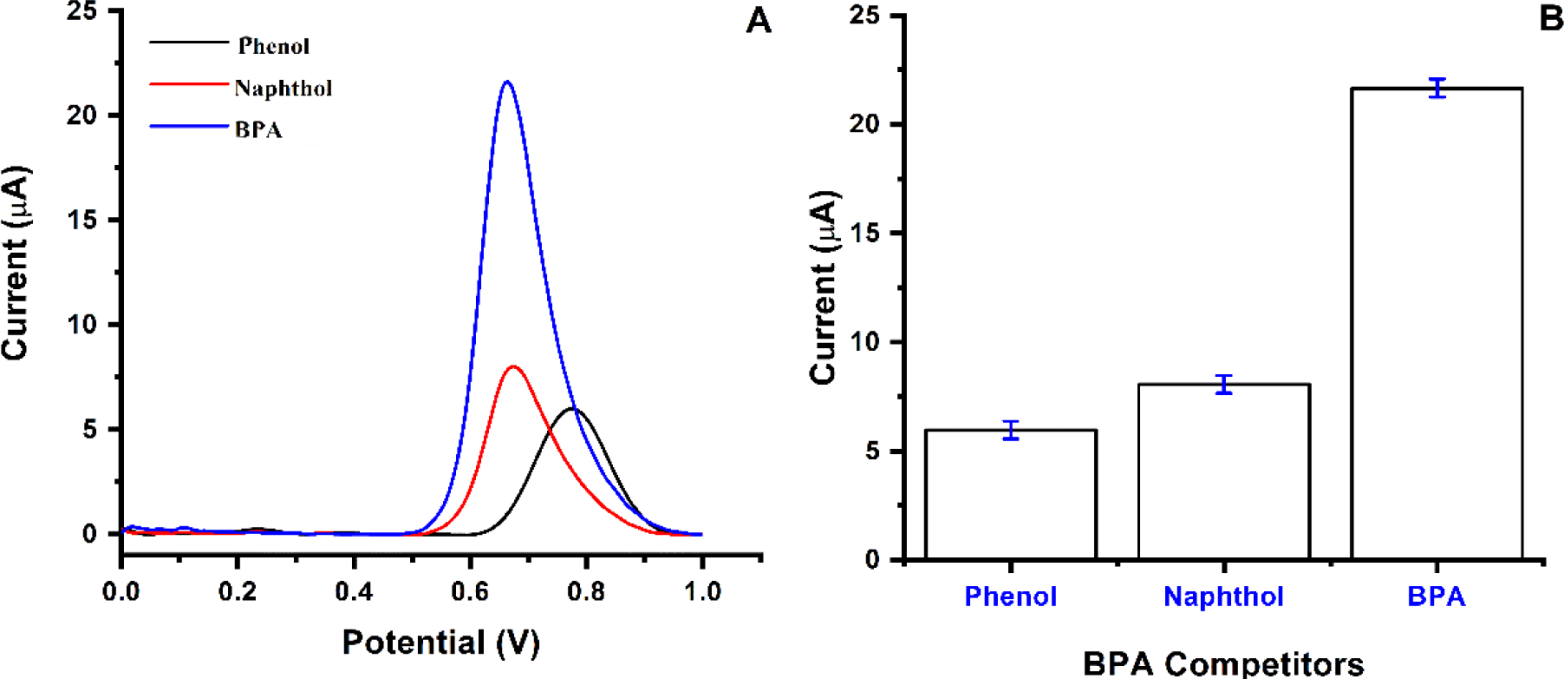
Selectivity study. (A) MIP-PGE response to BPA (7.5 μM), Naphthol (7.5 μM), and Phenol (7.5 μM) competitors; (B) bars representing the responses of MIP-PGE sensor (*n* = 3).

For this reason, the MIP-PGE sensor fabricated with a MAPA monomer containing phenylalanine can selectively detect BPA over other phenolic compounds, even at micromolar concentrations of BPA. Since the nature of the matrix is complex and interfering substances can be found in the matrix, it is necessary to achieve this selectivity to detect BPA accurately and reliably. Solutions containing BPA (7.5 μM), naphthol (7.5 μM) and phenol (7.5 μM) were prepared in 0.1 M acetate buffer solution at a pH of 4.8 to test the performance of the MIP PGE sensor. In addition, the same kinetic technique and procedure as before were used to analyze the behavior of the competitor molecules. The MIP PGE sensor was exposed to a BPA concentration of 7.5 μM to monitor the imprinting effect and determine the selectivity. In addition, competing molecules (phenol and naphthol) were used at a concentration of 7.5 μM to perform the competitive adsorption studies. Figure 8 demonstrates the results of this comparison, which shed light on the efficiency of imprinting and selectivity of the MIP PGE sensor for BPA detection.

By comparing the BPA signal response generated by the MIP-PGE sensor signal with the NIP-PGE sensor signal, the imprinting factor (I.F: 1.69) was calculated in order to evaluate the imprinting efficiency of the MIP-PGE sensor. Additionally, based on the relative selectivity values of the two sensors, it was found that the MIP-PGE sensor was 2.51 times more selective for phenol and 2.56 times more selective for 4-nitrophenol than the NIP-PGE sensor for BPA (Table 2).

**Table 2:** Selectivity and Relative Selectivity Coefficient Parameters of MIP-PGE and NIP-PGE Sensors

	MIP-PGE		NIP-PGE		
	Peak height (μA)	k	Peak height (μA)	k	k’
BPA	21.7		12.85		
Naphthol	8.0	2.71	12.10	1.06	2.56
Phenol	6.0	3.62	11.90	1.08	2.51

The selectivity coefficients (k = response for BPA/response for competitor) and relative selectivity coefficients (k’ = k_MIP_/k_NIP_) were calculated both for naphthol and phenol competitors. The resulting values indicate that the MIP sensor is 2.71 and 3.62 times selective for BPA compared to naphthol and phenol, respectively.

### 3.5 Repeatability of MIP PGE Sensor

The analytical detection by the polymer-modified electrode surface could be limited due to the decomposition that occurs on the surface of the imprinted electrode during the regeneration steps or measurements. Thus, the imprinted cavities on the surface of PGE intended for BPA could be affected and be less effective. As a result, the efficiency of the modified electrode can be impaired, and the analyte’s accuracy detection is hindered. Repeated analyses should be performed to ascertain the results for BPA detection. The experimental work should be done under the same conditions to minimize discrepancies. Measuring the BPA concentration at a precise interval, for example, hourly, over a period of time, and comparing the obtained results could be useful in determining the electrode capacity for the number of measurements for each electrode.

**Figure 9:**
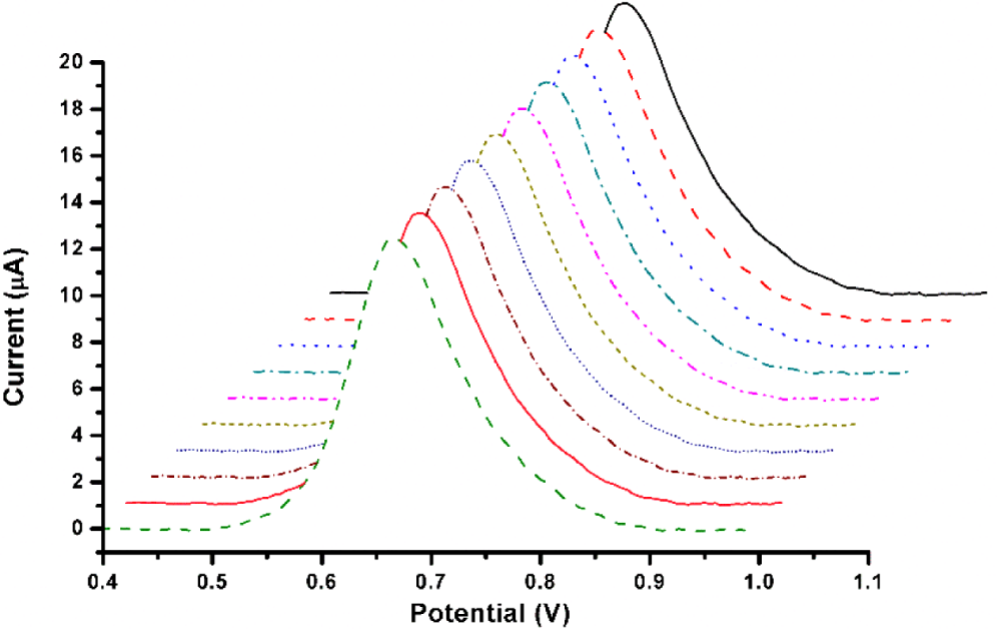
Repeatability study of the MIP PGE sensor. The response of the sensor electrode was for BPA (6.0 μM). The measurements were repeated 10 times (*n* = 10).

To conclude that the electrode has good repeatability, the results show consistency and reproducibility. The repeatability of the MIP-PGE sensor was determined using 6.0 μM BPA solution. Figure 9 shows the response of the MIP PGE sensor, projected as a potential in relation to the applied current. Generally, a RSD value of less than 5% indicates that the analytical analysis is precise and reproducible in most studies.^57^ The RSD% obtained for the 10 consecutive analyses demonstrates the exceptional reproducibility and reusability of the method, which is less than 1%, used to confirm the MIP PGE sensor’s three-dimensional stability. The consistency observed when performing multiple analyses shows that the MIP PGE sensor has the potential to robustly and reliably detect BPA in various applications. The stability of the MIP PGE sensor was tested for the detection of BPA by immersing the sensor in a BPA solution with a concentration of 6.0 μM. The DPV method detected BPA in 10 measurements (*n* = 10). By immersing the electrode in a desorption solution consisting of methanol and acetic acid in a ratio of 1:9 (v/v), desorption was achieved between the individual measurements. The equation below was used to evaluate voltammograms obtained from the ten measurements.
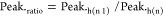
1

In the eq 1, the peak height is denoted by “h”, and “n” refers to the number of experiments performed. Based on the experimental outcomes, a relative standard deviation value was determined as 0.3189% for BPA, which was accomplished for the current (I) signal, which signifies the high stability of the electrode. The sensor’s response was obtained 10 times for the same BPA concentration of MIP PGE sensor with high efficiency since the RSD value is less than 1%, which means that MIP-PGE can be reused 10 times without loss of affinity and efficiency.

### 3.6 BPA Determination in Drinking Water Samples

**Figure 10:**
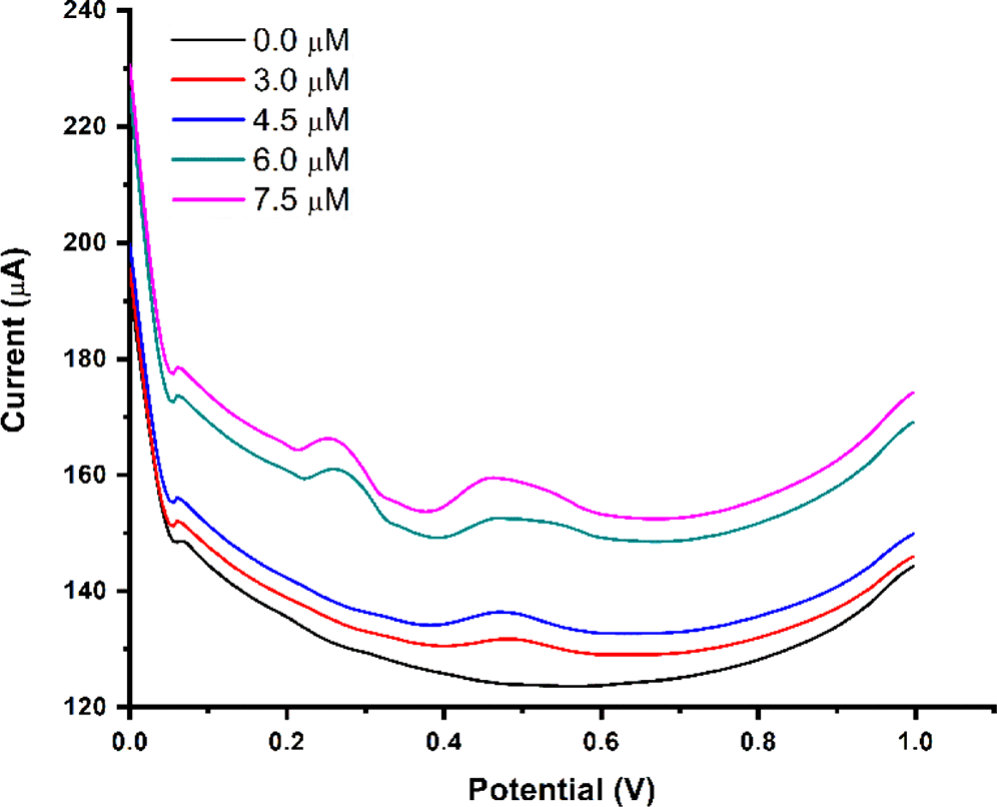
Voltammograms of spiked drinking water samples ranging from 0.0 μM to 7.5 μM.

The synthesized MIP PGE sensor was developed to specifically recognize BPA molecules from other molecules with similar functional groups (−OH groups) in drinking water samples. Moreover, the BPA molecules can be determined with high sensitivity and selectivity. Although BPA molecules have been directly detected in drinking water samples using electrochemical methods, various problems have been observed in several studies.^25,58^ For example, an increased background current and a high limit of detection due to a high overpotential in the oxidation of the phenolic hydroxyl group in the structure of BPA were observed on conventional electrodes.^59,60^ In addition, it has been observed in previous studies that oxidized BPA agents can easily adhere to the electrodes and appear as a thin film, leading to passivation of the proposed electrodes and thus reducing the sensitivity of the successful measurements. In this study, the MIP PGE sensor was held in drinking water samples spiked with different concentrations of BPA solutions prepared in acetate buffer solution with a pH of 4.8 to reach final concentrations of 3.0, 4.5, 6.0, and 7.5 μM. Figure 10 shows no voltammetric response in the nonspiked drinking water sample. In other words, no peak height was observed when the drinking water sample was analyzed without BPA solution.

Furthermore, the change in peak height observed in Figure 10 is due to the increase in the different BPA concentrations added to the drinking water samples. The lowest peak height was observed when 3.0 μM of BPA was added to the drinking water sample. Consequently, the developed BPA-imprinted MIP PGE sensor was easy to use, remarkably selective, fast responding, and sensitive for the detection of BPA in drinking water samples. MIP PGE, which contains an amino acid in its structure, was developed without labeling processes and complicated coupling methods to detect BPA molecules. These unique properties, in particular, make the sensor a practical and attractive device for measuring BPA levels efficiently and accurately.

To date, various analytical methods have been developed to quantify BPA, including enzyme-linked immunosorbent assays (ELISA), gas chromatography with mass spectrometry (GC-MS), liquid chromatography with mass spectrometry (LC-MS), fluorescence spectroscopy (FL), surface-enhanced Raman spectroscopy (SERS) and high-performance liquid chromatography (HPLC), and innovative sensor technologies.^7,36,61,62^ While several methods have shown excellent detection limits and selectivity for BPA, they are expensive, require pretreatment and sample preparation.^61,63^ Particularly, sample preparation often involves expensive instrumentation and several time-consuming steps such as purification, extraction and separation.^2,7^ One study highlighted that a derivatization procedure is required for the detection of BPA using some analytical techniques such as GC-MS.^64^ For example, endocrine disruptors is determined in water after derivatization with *N*-methyl-N-(tertbutyldimethyltrifluoroacetamide) by GC-MS.^65^ According to the literature, the detection limit for instrumental analyses can reach 4–6 ng L^–1^. However, these methods are not suitable for in situ detection and batch determination, which require complex pretreatment steps.^65^ Although immunochromatographic assays and ELISA frequently detect BPA, they lead to false positive results and incomplete quantification. On the other hand, despite their drawbacks, immunoassays are popular for BPA screening due to their speed and simplicity.^7^ Moreover, the current methods are to be often only able to detect simple samples with low matrices such as baby bottle extracts and spring water. However, it is still very challenging to perform direct analysis of BPA in complex matrices such as biological samples, soil leaching solution and environmental water. Therefore, fast and advantageous methods should be developed for the direct analysis of complex matrices.^61^

On the other hand, electrochemical sensors are widely utilized in environmental, food, and healthcare analysis due to their high specificity and sensitivity. They offer advantages such as being inexpensive, fast, environmentally friendly, and capable of real-time detection under in situ conditions.^66^ Notably, BPA has electrochemical properties due to a phenolic group in its chemical structure. However, it does not show a strong response, and it becomes more challenging to determine BPA using the electrochemical method.^1^ Besides, conventional sensors are unlikely to detect BPA effectively due to their low selectivity for this compound.^67^

In this study, a sensor for bisphenol A (BPA) was developed based on DPV detection using MIPs and gold nanoparticles. In detail, the PGE electrode used in this study is an example of novel kind of electrode and has been widely used in recent years.^68,69^ A wide range of analytes are identified by different voltammetric methods that uses PGE.^17^ PGE has many advantages over other carbon-based electrodes such as surface polishing, low cost and disposibility.^21^ In addition, the surface can be modified easily and it has high surface area and electrochemical reactivity.^22^ Second, by using MAPA on the electrode’s surface, the sensor’s selectivity is significantly increased, which improves the detection of BPA even at low levels in the drinking water samples. Furthermore, in order to increase the sensitivity of the detection method a various of materials such as carbon materials,^70^ ionic liquids,^71^ aptamers,^61^ metals and covalent organic frameworks,^72^ MIPs,^73^ metal or metal nanoparticles^74^ are used to modify the working electrode. In this study, the PGE electrode was coated with N-methacroyl-(L)-cysteine methyl ester and N-methacryloyl-(L)-phenylalanine methyl ester. More importantly, AuNPs and MIPs were used in the modification of the PGE electrode. Particularly, AuNPs increased the electroactivity and surface area of the developed sensor imprinted with BPA and the sensitivity of the electrode is enhanced with MIPs.

Although enzymes and antibodies are used as recognition elements in several studies, MIPs have several advantages over these elements. For example, they can be synthesized easily and in a short time, are less expensive and are chemically and physically more stable.^35,75^ These advantages of MIPs overcome many disadvantages of alternatively synthesized recognition materials. Since the MIP PGE sensor can accurately detect BPA molecules in drinking water samples, it can be used to detect BPA in complex environmental matrices.

In recent years, BPA has attracted great attention because it is produced in large quantities, is widely used in industry, and is widely dispersed in the environment. In particular, this toxic substance can contaminate water, beverages, and food. It is also an endocrine disruptor that has estrogenic properties and can mimic estrogen. Exposure to BPA has been linked to various diseases, including obesity, cancer and type 2 diabetes,^1,8,76^ Therefore, detecting BPA in environmental samples such as drinking water, seawater, lake water, and other complex matrices such as biological fluids could open up new opportunities to assess the impact of BPA exposure on human health. Especially, water pollution is still a growing health and environmental problem all around the world. To date, expensive and time-consuming methods are used for BPA analysis,^13,77,78^ Therefore, it is necessary to develop sensitive methods to determine toxic compounds such as BPA. In the literature, in a study, a rapid, environmentally friendly method was developed for the determination of BPA concentrations in urine samples using the liquid chromatography–mass spectrometry (LC-MS^2^) method in combination with porous organogel materials. It has been indicated that the methods mentioned in this study were only suitable for the measurement of BPA and not for the detection of BPA and its analogues in a single run.^79^ In another study, LC-MS^2^ and gas chromatography mass spectrometry (GC-MS) were used to determine and confirm BPA and other endocrine disruptor chemicals (EDCs). In this particular study, both methods required sample preparation, and LC-MS^2^ was unable to detect nonylphenol mixtures at low concentrations. However, GC-MS was able to determine all analyzed compounds with good accuracy and repeatability. The same researchers also pointed out that GC is less frequently used for the analysis of BPA compared to LC. There are several reasons for this, such as the long sample preparation and derivatization steps for the analyte, as well as the purification procedure, which makes the method inefficient and impractical for testing a larger number of samples.^80^ In another study, trace BPA was detected by a method based on a diazotization coupling reaction combined with liquid chromatographic method with ultraviolet detection (LC-UV) and the LOD value was determined to be 0.15 μg/mL (0.657 μM).^81^ Moreover, in a different study, indirect and direct competitive methods were developed and compared for the detection of BPA in human urine samples. Based on the results of this study, direct competitive ELISA was found more specific and sensitive compare to indirect competitive ELISA and the LOD values were reported as 0.03 and 0.08 ng/mL (0.131 μM and 0.350 μM) respectively.^7^ Lastly, other study used a lateral flow strip which was developed by conjugating gold nanoparticles (AuNPs) with anti-BPA antibodies. This method was detected BPA with a LOD value of 5 ppb (21.9 μM).^7^

In our study, BPA was sensitively detected by an electrochemical sensor based on gold nanoparticle-doped MIP. The developed sensor was effectively used for the determination of BPA in the presence of potential interferences. The LOD and LOQ values, linear range, sensitivity and other measurements were demonstrated in Table 1. Importantly, among the measurements, the LOD value in particular measures the sensitivity of a detection method, and the sensitivity increases as the LOD value decreases. The detection of BPA using the MIP PGE sensor has produced the lowest LOD compared to other research studies in Table 3. For example, a study using poly(glutamic acid)-modified PGE reported a LOD of 0.37 μM for BPA detection using an electrochemical method^77^ whereas in our study using the MIP PGE sensor, an LOD value of 0.1610 μM was obtained, suggesting that the technique used in our study is more sensitive. In another study using a different type of electrode but the same method as in this study (DPV), a LOD value of 0.290 was determined,^82^ which is higher than the LOD value in our study (0.1610 μM), Furthermore, in a different study, a different type of electrode (cathodically pretreated BDD) was used for BPA detection with an LOD value of 0.210 μM by the same method used in this study (DPV).^83^ Lastly, a different study which used an electrochemical sensor developed based on glassy carbon electrode (GCE) modified by a reduced graphene oxide-silver/poly-l-lysine nanocomposite detected BPA with a LOD value of 0.54 μM.^4^

**Table 3:** Comparison of the Developed Method with Some of the Previously Documented Methods

Method	Electrode	LOD (μM)	References
CV& Amperometry	PEDOT/GCE	90–410 and 40–410	(84)
Amperometry	Laccase–thionin–carbon black-modified	0.2	(85)
CV& DPV	PGA/PGE	0.37	(77)
DPV	ITO	0.290	(82)
Amperometry	EC(CYP2C9-PAM/GCE)	0.58	(86)
DPV	Cathodically pretreated BDD	0.210	(83)
CV and LSV	Zinc Oxide/Graphene Oxide modified carbon paste electrode (ZnO/GO/MCPE)	0.209	(87)
ASV	Modified Fly Ash carbon paste electrode (MFA/CPE)	0.31	(88)
DPV	RGO-Ag/PLL/GCE	0.54	(4)
DPV	MIP PGE Sensor	0.1610	This Study

Overall, compared to other studies shown in Table 3, our study’s LOD value for BPA detection with the MIP PGE sensor is significantly lower. In addition, a high reproducibility and selectivity for BPA detection was achieved in our study with the MIP PGE sensor. The method used in this study to detect BPA is specific and reliable, which is very important in practical applications where reproducibility and specificity are crucial. In summary, this technique has proven highly sensitive, selective and reproducible, making it a promising method for detecting BPA in various practical applications such as ecotoxicity studies. In addition, the detection of BPA in the presence of analogous structures such as phenol and naphthol has been successfully performed with the MIP PGE sensor, which has high sensitivity and selectivity.

## 4 Conclusion

In conclusion, in this particular study, BPA was successfully detected with a low detection limit using a MIP PGE sensor. The detection of competitors with BPA also showed selectivity. The same method was also used to detect BPA in drinking water samples. The electrodes showed high stability and repeatability in the different DPV detections of BPA. Therefore, the newly developed sensor can be used for the detection of BPA in various environmental analyses due to its high sensitivity, simplicity, low cost, and ecological congeniality. Importantly, during the development of the electrode, since imprinted cavities were created on the surface of the electrode, resulting in a structure with binding sites that have high specificity for the template molecule, an electrochemical MIP PGE sensor can specifically detect BPA. Since this method showed high specificity, BPA can be detected at low concentrations, making the MIP PGE sensor a promising alternative in environmental studies and practical applications.

Furthermore, compared to other naturally occurring biological receptors, it is easier and cheaper to synthesize an MIP PGE sensor to detect BPA. In addition, the selectivity of the designed electrode with increased selectivity and sensitivity for the BPA molecule was also measured in the presence of analogous molecules (phenol and naphthol). Moreover, BPA was successfully detected in drinking water samples by MIP PGE. The detection of BPA molecules with the existence of BPA competitors such as phenol and naphthol was achieved with a value of less than 0.17 μM. No additional modifying agents, such as spacer arms for immobilizing ligands, were required to detect BPA. The MIP PGE sensor detected BPA quickly with an LOD of 0.1610 μM and high sensitivity. Based on the results, the MIP PGE shows the highest selectivity and sensitivity among all electrode types mentioned in this study. In summary, a BPA-imprinted electrochemical sensor was developed, and BPA was detected in real-time with high sensitivity in aqueous solution and drinking water samples. This advanced approach can detect BPA in drinking water samples with high selectivity and sensitivity.
